# Comparative effects of high-intensity interval training at low and moderate altitudes on 5000-m performance and perceptual responses: A randomized controlled trial

**DOI:** 10.1016/j.jesf.2026.200460

**Published:** 2026-02-18

**Authors:** Sisay Fentaw, Tefera Tadesse, Zerihun Birhanu

**Affiliations:** a Sport Academy, Bahir Dar University, Bahir Dar, Ethiopia; b Sport Science Department, Debark University, Debark, Ethiopia; c Educational Development and Quality Center, University of Global Health Equity, Kigali, Rwanda

**Keywords:** Long-distance running performance, Psychophysiology, Pacing adaptation, Hypoxia, High-intensity interval training, Altitude training, Perceived exertion

## Abstract

**Background/objectives:**

Hypoxia compromises training quality, yet high-intensity interval training (HIIT) performed under hypoxia may elicit greater benefits than does normoxic HIIT. However, its effects on pacing and perceived exertion remain underexplored, particularly in moderate-altitude distance runners. This study aimed to compare the efficacy of HIIT performed at low (∼1220 m) versus moderate (∼2850 m) altitudes on pacing and rated perceived exertion (RPE) during a 5000 m race.

**Methods:**

Forty-two moderate-altitude inhabitant runners (men/women, 23/19) were randomly allocated into the HIIT groups at ∼2850 m (HIIT2850m, n = 14) and at ∼1220 m (HIIT1220m, n = 14), and the control group at ∼2850 m (CG2850m, n = 14). The HIIT intervention was completed by the HIIT2850m group at moderate altitude and the HIIT1220m group, which travelled to low altitude for 8 weeks with 2 sessions.wk^−1^. Each session consisted of 4 × 4 min intervals at 100% velocity at maximum oxygen consumption (vVO_2_ max, determined at their respective training sites), with 3 min recovery at 70% vVO_2_ max intensity. Before and after the intervention, 5000 m races were performed in a matched group on a 400 m track at ∼2850 m and every km speed and RPE were recorded.

**Results:**

The results indicated that the participants used a similar parabolic reversed-J shaped strategy, with significant 5000-m time improvements observed in both the HIIT1220m (Δ: 20.1 ± 23.7 s, p = 0.007) and the HIIT2850m (Δ: 16.7 ± 25.9 s, p = 0.003) groups. A significant main effect of time (except at the 2^nd^ km, p = 0.693) and a main effect of group (at the 1^st^ km, p = 0.034 and 5^th^ km, p = 0.044) were observed in pace, along with a significant group-by-time interaction in all km segments. Compared with the CG2850m, both interventions demonstrated significantly greater speeds at the 1^st^ and 5^th^ km and lower RPE, with greater changes in the HIIT1220m group.

**Conclusion:**

The results suggest that HIIT at both altitudes improved performance and RPE, with quality training at low altitudes producing greater benefits, offering strategic insights for athletes and coaches.

## Highlights

• High-intensity interval training (HIIT) improved 5,000 m time, pacing speed, and reduced perceived exertion.
• Lower perceived exertion reflected better intensity tolerance and pacing regulation, resulting in faster 5,000 m times.
• HIIT performed at low altitude elicited greater benefits in pacing and perceptual responses than did at moderate altitude.

## 1 Introduction

Athletes devote themselves to various type of training under diverse intensities and environments. Researchers have also continuously examined several training methods to optimize pacing and reduce perceived effort. These efforts aimed to optimize training and achieve optimal performance. In fact, experienced athletes are more aware of the timing and mechanisms used to economically distribute energy in the race, known as pacing. As such, training intensity ranges from near maximal to supramaximal intensity has a significant influence on pacing strategy and perceived exertion.^1^^,^^2^ Among the infinite training methodologies, hypoxic conditions and high-intensity interval training (HIIT) are popular for efficiently changing performance within a short time, either separately or in combination.^3^^,^^4^

HIIT is a form of intensive endurance training performed either near or at maximum oxygen consumption (VO_2_ max) or ‘hard’ to ‘very hard’ rated perceived exertion (RPE) of ≥15 on the 6–20 Borg scale, which results in oxidative metabolism and sustained maximal aerobic speed.^5^, ^6^, ^7^ Studies have shown that 10–30% of the training distribution of macrocycles employs high-intensity training.^8^^,^^9^ In turn, it improves endurance performance and enjoyment more than other training methods do.^10^^,^^11^ It also helps prevent training plateaus and develop a tactical pace at a sustained VO_2_ max, enabling athletes to maintain a faster pace throughout the race.^12^ Thus, HIIT is considered an essential component of higher-volume training programs for successful long-distance performance.^8^ In particular, HIIT performed at lower altitudes results in improved performance and reduced RPE.^13^^,^^14^

However, HIIT under hypoxia conditions may compromise training quality by reducing absolute speed due to limited oxygen availability. This reduced VO_2_ max and speed can increase the RPE and pacing effort.^15^ The increased RPE directly regulates exercise intensity and pacing,^16^ which disrupts pacing, leading to conservative running and increased fatigue. Collectively, these conditions impair training and competitive performance. Nevertheless, HIIT under hypoxia has also been shown to aid endurance athletes in better tolerating fatigue and maintaining a more even pacing strategy in races.^17^^,^^18^ With respect to its effectiveness, studies have reported conflicting findings. On the one hand, when HIIT is performed under hypoxia (live-low train-high, LLTH) compared with normoxia (live-low train-low, LLTL) conditions, performance time improvements are greater.^3^^,^^19^^,^^20^ In contrast, no significant difference were detected between the protocols.^21^^,^^22^ On the other hand, when HIIT was performed at lower altitudes, both the live-high train-low (LHTL) and LLTL protocols were significant, with greater improvement observed in the LHTL,^23^ but no specific effect was found at 3 km.^24^ Furthermore, a four-week HIIT intervention does not necessarily change the pacing strategy, performance time, or RPE during a 5 km trial of endurance runners,^25^ which may reflect an insufficient training duration to elicit meaningful adaptations. Bejder & Nordsborg^26^ concluded that when statistical and methodological rigor were properly applied, LHTL had no effect on performance.

While the benefits of HIIT and high-altitude training are widely recognized, the specific influences on pacing and perceived exertion remain insufficiently explored. Although evenly distributed pacing has been demonstrated to improve endurance performance time,^27^^,^^28^ examining running event pacing strategies remains limited compared with other endurance sports.^29^ Notably, among endurance athletes residing at moderate altitudes who complete almost all training at this altitude, a reduced pacing speed, along with high exhaustion during races, is common. In addition, to the authors’ knowledge, no studies have systematically examined whether HIIT performed at low or moderate altitudes can influence performance in this population. The rationale for conducting HIIT at lower altitudes is that reducing hypoxic stress may allow higher-quality sessions and greater absolute intensities, which could subsequently influence performance and RPE.

This area has attracted increasing interest because of its multifaceted significance. Theoretically, moderate-altitude resident long-distance runners constitute an underrepresented population that warrants further research.^4^ Methodologically, this approach facilitates a better understanding of athletes’ adaptations regarding pacing tactics and perceived effort across different segments of a 5000 m race. In practice, endurance running athletes, coaches and researchers also have the opportunity to understand the possible underlying mechanisms and refine training strategies to optimize performance. In light of the existing evidence and identified gaps, this study was initiated to compare the effects of eight weeks of HIIT performed at low (∼1220 m) and moderate (∼2850 m) altitudes on the 5000-m performance and perceptual response of long-distance runners living at moderate altitudes. Given the greater capacity to perform quality training at low altitudes, we anticipate that, compared with HIIT conducted at moderate altitudes, HIIT performed at low altitudes would increase pacing speed at the beginning of the first km, followed by speed reductions during the 2^nd^, 3^rd^, and 4^th^ kilometers and a subsequent increase in the final km, resulting in reduced (faster) time. We also hypothesized that overall RPE would be lower in both intervention groups than in the control group.

## 2 Methods

### 2.1 Participants

The included participants were (1) specialized at 3 km to marathon running; (2) trained for ≥3 years; (3) were chronological in age ≥18 years; (4) did not perform systematic training during a transition period; (5) had at least a history of regional competition; and (6) were not trained or exposed at low altitudes (<1500 m) within the previous three months for more than 48 h. On the other hand, participants with recent illnesses or injuries that limit training and racing, such as heat allergy, epistaxis, malaria, gastrointestinal distress, pregnancy, and anaemia, were excluded.

### 2.2 Sample size determination

A priori analysis was performed on the basis of Park et al.^20^ 3000-m time trial interaction effect (η^2^ = 0.097), resulting in the adoption of Cohen's f = 0.34. Accordingly, we determine the required sample size via power analysis for a repeated-measures analysis of variance (ANOVA) with a within-between interaction (three groups with pretest‒posttest measures), α = 0.05, power = 0.95, effect size = 0.34, the correlation among repeated measures was set to 0.5, and ε = 1 was conducted in G∗Power 3.1.9.7. As a result, 13 subjects per group were required to detect the effect size. A possible 10% dropout rate was also expected, with 5 more subjects added for matched distributions among groups, resulting in 16 subjects per group (n = 48). After completion of the study, a post hoc sensitivity analysis was conducted via G∗Power to determine the minimum detectable effect size and achieve statistical power. It was performed via the final actual sample size, statistical design, and observed effect sizes.

**Fig. 1:**
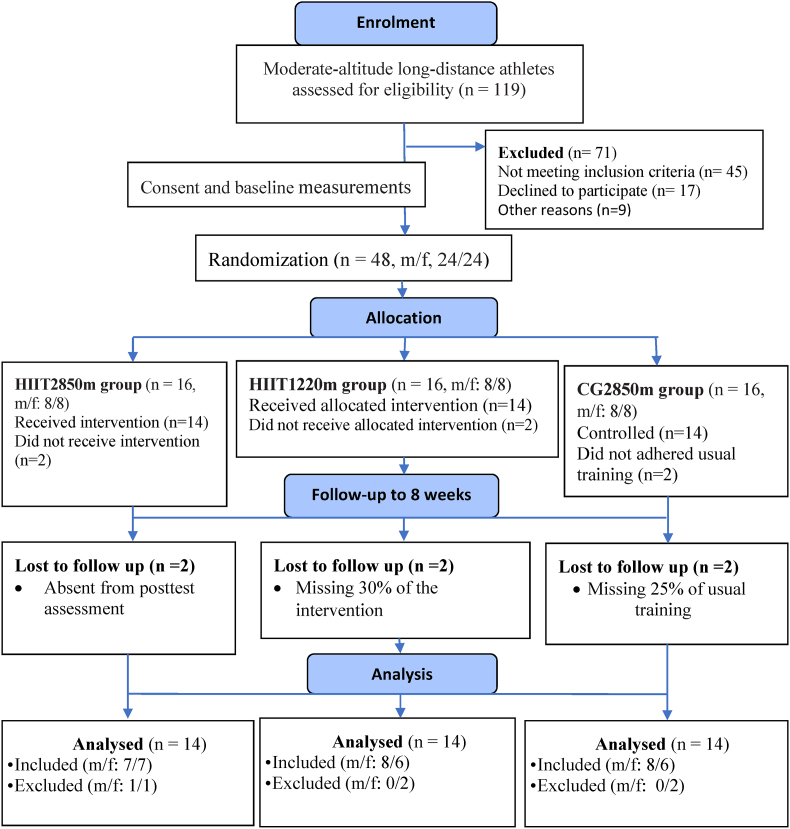
Flowchart for the participants in the study process.

Among the 119 running inhabitants initially screened, 71 were excluded, and a total of 48 distance runners (male/female, n = 24/24) who lived and trained at ∼2850 m in Debark Town, were recruited from local athletics clubs during the preparation period. Following verbal and written explanations of the study purpose and process, the participants provided written consent to participate. The first and third authors conducted the coding and stratification. The second author, who was situated outsite, generated the allocation sequence via stratified block randomization and sequential numbering as displayed in Fig. 1. The participants were then assigned to three groups: the HIIT group at ∼2850 m (HIIT2850m, n = 16, m/f, 8/8), the HIIT group at ∼1220 m (HIIT1220m, n = 16: m/f, 8/8), or the control group at ∼2850 m (CG2850m, n = 16: m/f, 8/8). However, six (m/f, 2/4) participants dropped out because >20% usual and intervention training adherence (n = 4) and posttest measurements (n = 2) were missed.

The outcome assessors and data analysts were blinded to the group allocation; however, the participants could not be blinded because of the practical realities of conducting training at two different natural locations. The participants were instructed not to disclose their group assignment across measurements. The final analysis (n = 42) included HIIT2850m (n = 14, m/f, 7/7), HIIT1220m (n = 14: m/f, 8/6) and CG2850m (n = 14: m/f, 8/6), as presented in Fig. 1. The overall characteristics of the participants, stratified by sex within each group, are presented in Table 1, Table 2.

**Table 1:** Subject baseline anthropometric, training and performance characteristics based on sex distribution at inclusion.

Parameter	Sex			Group					
	male	female	Overall	HIIT2850m		HIIT1220m		CG2850m	
				male	female	male	female	male	female
Size (n)	23	19	42	7	7	8	6	8	6
Age (yr)	24.6 ± 4.5	19.3 ± 1.5	22.2 ± 4.3∗	25.6 ± 5.3	18.9 ± 0.7	24.9 ± 4.8	19.7 ± 2.3	23.4 ± 3.5	19.5 ± 1.5
Running age (yr)	7 ± 2.6	5.2 ± 1.2	6.2 ± 2.3∗	7.6 ± 3.1	5 ± 0.8	7 ± 3	5.5 ± 1.8	6.5 ± 1.9	5.2 ± 1.2
Body mass (kg)	53.2 ± 2.7	45 ± 4	49.5 ± 5.3∗	54 ± 2.4	45.4 ± 3.7	53.3 ± 3.2	45.6 ± 6.3	52.5 ± 2.5	43.9 ± 1
Height (m)	1.69 ± 0.04	1.58 ± 0.04	1.64 ± 0.07∗	1.7 ± 0.02	1.6 ± 0.03	1.59 ± 0.04	1.6 ± 0.07	1.67 ± 0.05	1.59 ± 0.04
BMI (kg/m^2^)	18.7 ± 0.8	18.1 ± 1.4	18.4 ± 1.2	18.7 ± 0.4	18.7 ± 1.7	18.7 ± 0.5	18 ± 1.5	18.9 ± 1.2	17.5 ± 0.95
VO_2_ max (ml/kg/min)	68.8 ± 4.9	56.4 ± 1.7	63.2 ± 7.3∗	69 ± 5.4	56.6 ± 2.1	68.8 ± 5.3	56.2 ± 1.6	68.5 ± 4.5	56.3 ± 1.7
5-km time (min)	17 ± 1.2	19.3 ± 1.5	18 ± 1.7∗	16.7 ± 1.6	19.7 ± 1.6	17.1 ± 0.5	19 ± 1.2	17.2 ± 1.4	19 ± 1.5
Frequency (days.wk^-1^)	7 ± 1.4	6.6 ± 1.2	6.8 ± 1.3	6.7 ± 1.1	6.4 ± 1	7.1 ± 1.1	6.3 ± 1.2	7.1 ± 1.8	7 ± 1.4

**Note**: Data are presented as the mean ± SD, ∗ significant at p < 0.05.

Abbreviations: HIIT2850m, high-intensity interval training at moderate altitude; HIIT1220m, high-intensity interval training at low altitude; CG2850m, control group at moderate altitude; BMI, body mass index; VO_2_ max, maximum oxygen consumption.

**Table 2:** Baseline anthropometrical, training, and performance characteristics of the subjects in each group.

Parameter	Group			HIIT2850m vs HIIT1220m p value	HIIT2850m vs CG2850m p value	HIIT1220m vs CG2850m p value
	HIIT2850m n = 14	HIIT1220m n = 14	CG2850m n = 14			
Age (yrs.)	22.2 ± 5	22.6 ± 4.6	21.3 ± 3.4	0.911	0.675	0.575
Running age (yrs.)	6.3 ± 2.6	6.4 ± 2.6	5.9 ± 1.7	0.944	0.623	0.573
Body mass (kg)	49.7 ± 5.4	49.9 ± 6	48.8 ± 4.8	0.818	0.531	0.75
Height (m)	1.63 ± 0.08	1.65 ± 0.07	1.63 ± 0.06	0.913	0.686	0.784
BMI (kg/m^2^)	18.7 ± 1.2	18.4 ± 1.1	18.3 ± 1.3	0.724	0.757	0.998
VO_2_ max (ml/kg/min)	62.4 ± 7.3	62.4 ± 7.7	62.4 ± 7.2	0.998	0.99	0.992
5-km time (min)	18.2 ± 2.1	18.3 ± 1.5	18.3 ± 1.8	0.947	0.942	0.992

The data are presented as the means ± SDs. The groups were as follows: HIIT2850m, high-intensity interval training group at moderate altitude; HIIT1220m, high-intensity interval training group at low altitude; and CG2850m, a control group at moderate altitude. The parameters used were BMI, body mass index; VO_2_ max, maximum oxygen consumption.

### 2.3 Experimental design

An 8-week randomized control trial design was employed, to reduce differences and ensure balanced distribution across groups, on the basis of sex, 5000 m time, and event specialization. The study protocol was carried out according to the Helsinki Declaration and was approved by the Sport Academy Research Ethics Committee at Bahir Dar University (Pro. No: IRERC 06/2024). The contents adhered to the CONSORT guidelines for reporting randomized controlled trials.

The study was conducted (September 26–November 20, 2024) during the specific preparatory phase of the athletes’ cross-country macrocycle (23 weeks), immediately following seven weeks of general conditioning (August 8–September 25). This timing coincided with significant increases in the volume and intensity of the macrocyle training plan when the athletes were transitioning from building an aerobic base to focusing on performance-specific adaptations. Daily training loads were monitored by training diaries and coach training plans (quantified volume in kilometers, intensity in ratings, duration in time and type of training in kind), which the research team reviewed for consistency and adherence.

The intervention groups performed extra HIIT sessions at two different altitude locations, i.e., the HIIT2850m group completed the sessions at their residences in Debark Town (∼2850 m), whereas the HIIT1220m group travelled to a low-altitude site for each HIIT session. These sessions were carefully scheduled as two nonconsecutive days to allow sufficient time to recover and adapt.

The intervention duration and frequency were selected on the basis of evidence demonstrating that HIIT is a time-efficient and powerful training stimulus capable of inducing adaptations within a relatively short intervention time, even in trained endurance athletes.^6^ Several studies have reported significant improvements in aerobic capacity and running performance following 4-week^25^^,^^30^^,^^31^ and 6-week^32^ periods of HIIT performed twice.wk^−1^, in both added and replaced conditions with conventional training programs. Most importantly, two HIIT sessions.wk^−1^ appear to be an effective strategy, with no clear advantage gained from increasing the frequency to 3 sessions.wk^−1^.^32^ Accordingly, the present study was designed to supplement the participants’ usual training with structured HIIT sessions to reflect real-world training practices.

### 2.4 Application of HIIT in training macrocycles

The training plan was organised into a single-peak macrocycle designed to culminate in one main cross-country competition. The macrocycle comprises three main periods: preparation (including general and specific phases), competition (precompetition and main competition phases), and transition. In endurance running, the distribution of training load typically follows an inverse relationship between volume and intensity, where a progressive increase in volume and intensity during the early stages is accompanied by a gradual reduction in volume and a corresponding increase in intensity toward the later stages of the season to optimize performance and minimize fatigue. Within this framework, HIIT sessions were strategically integrated into the microcycle via an undulating step loading pattern, scheduled on two nonconsecutive days to alternate among moderate, low, and regeneration intensity loads.

#### 2.4.1 Determining intensity

Training intensity was monitored through the predicted velocity at VO_2_ max (vVO_2_ max), which was derived from a 5-min maximal aerobic speed test. This test estimates that the running velocity corresponding to the attainment of VO_2_ max starts to occur. While direct laboratory measurements represent the gold standard, their expensiveness and impracticality for large populations led to the development of field test predictive equations. The 5-min trial is the easy, quick and strongly correlates with the laboratory-measured vVO_2_ max (r = 0.94), supporting its validity for training prescription,^33^^,^^34^ in which athletes perform maximal efforts in the allotted time to cover more distances as fast as possible.

### 2.5 Training protocol

The experimental groups completed the well-established HIIT session (4 × 4 min of work intervals at 100% vVO_2_ max with 3 min of recovery at 70% vVO_2_ max), which consisted of 4 work interval bouts with a 4-min interval duration, interspersed with 3 min of active rest. Before and after the session, a 10-min warm-up consisting of continuous, interval runs and dynamic stretching and a 5-min cool-down with light jogging and static stretching were completed. While the HIIT2850m group performed the intervention at their residences (∼2850 m), the participants in the HIIT1220m group were transported to low altitudes (6:00–8:00 a.m.), which are located in the foothills of Mountains at Zarima Town, 38 km away from their residences. Prior to training and travel, all participants were instructed to adhere to their usual hydration and nutritional practices. During travel, to minimize fatigue and mitigate potential environmental confounders, the HIIT1220m group was permitted to consume water and carbohydrate snacks as needed. Upon arrival, participants were provided a 20–30-min passive rest to facilitate recovery before engaging in subsequent training.

At each altitude training site, the HIIT number of intervals, interval duration, and intensity during work and rest intervals remained constant throughout the intervention to ensure reliable training stimuli. The intensity was determined on the basis of each participant's vVO_2_ max taken at each training venue, in which participants were grouped on the basis of the equivalent vVO_2_ max for the intervention. Intensity matching was used to ensure that any observed effects were due to the true impact of the intervention, which ensured ecological validity, rather than differences in dose intensity. In each interval run, in addition to a verbal instruction, a whistle signal was also blown to alternatively utilize the 100% and 70% vVO_2_ max performance to complete efficiently. Accordingly, immediately after each interval of work and recovery, training intensity was continuously monitored by two experienced coaches and two sport training research experts in combination via vVO_2_ max, pace, and the Borg scale (6–20) to ensure that each participant work interval was at least 18 RPE and corresponding 100% vVO_2_ max pace, whereas active recovery intervals were maintained at 11–13 RPE and 70% vVO_2_ max.

### 2.6 Procedures

The subjects performed a 20-min warm-up and dynamic stretching for a 5000 m run, after a significant recovery day. A group of athletes (≤8), who have an equivalent of 5 km, race in a group paced to complete the best time records. The subjects were instructed to provide their RPE every km via verbal reporting of the Borg 6-20 RPE scale.^35^ The details of perceived feelings of tiredness and how to provide RPE scores were trained during HIIT and explained before the race warm-up for familiarization with the procedure. Each participant had a visible identification number and was assigned a trained data collector (among 4 coaches and 4 sport research experts).

#### 2.6.1 Measurements

The data were measured one week before and subsequently after the HIIT intervention at ∼2850 m between 8:30 and 10:00 a.m. under similar environmental conditions (temperature = 12.6–19 °C; wind speed = 7.9–10.7 km.h^−1^; air pressure = 1.002–1.007 atm; relative humidity = 60–65%) to minimize the time of day's circadian rhythm. The subjects were instructed to maintain their usual training, recovery, nutritional, and hydration habits and to abstain from new training and supplementation throughout the study period, as well as from intense training two days before the race. The participants completed a 5000 m race in the presence of a group of performance-equivalent runners to evaluate kilometer by kilometer pacing speed, RPE, and performance time. Each participant's split time (subsequently, the pacing speed was computed in km.h^−1^), and the RPE at each kilometer was recorded.

Following a warm-up, a 5000 m running test was performed on a 400 m outdoor track. Athletes were instructed to achieve the shortest possible time. The collective running race was performed by a small group of subjects whose equivalent time was placed in the same starting position. The start was given as usual in the competition at the start line for the running race. The subjects were asked to run at individual paces as much as possible for best 5000 m time. Wearing watches during the run was not allowed, whereas 400 m split times were verbally communicated to the participants. Eight experienced timers, four of whom were athletes’ coaches and four of whom were assessors, were used. Two timers were assigned to one participant, and the mean values were then used for analysis.

##### 2.6.1.1 Performance time

The 5000 m completion time was recorded in a head-to-head race with a group of athletes who had equivalent performance levels before the intervention. Accordingly, the total time to complete the 5000 m was recorded to the closest 0.01 s as the athletes crossed the finish line.

##### 2.6.1.2 Pacing speed and strategy

During the race, every kilometer split time was recorded. Consequently, the speed of each kilometer (km.h^−1^) was determined to establish the pacing strategy of each athlete. Each kilometer pace (min.km^−1^) was also calculated.

##### 2.6.1.3 Perceived exertion

The Borg 6-20 RPE scale was used to assess participants' overall level of perceived effort during each kilometer completion of the 5000 m run. The participants were required to report their RPE immediately after completing each kilometer via the 15-point Borg category ratio (CR 6--20) via standardized verbiage.^36^ This scale is widely used to assess perceived intensity of effort and has 8 categories ranging from 6 to 7 (no effort at all) to 20 (maximal effort).^35^ Before running, the participants were familiarized with the scale by explaining the characteristics of each rating, what feelings should evolve, and how to rate a single score depending on these representations.

### 2.7 Statistical analysis

The data are presented as the means ± standard deviations for comparisons. Independent samples t tests were employed to identify baseline differences between groups. All the data were verified for homogeneity of variance and normality via Levene's test and the Shapiro‒Wilk test, respectively (p > 0.05), to ensure analysis validity. However, the sphericity assumption was not required because of the two point measures, which are inherently satisfied. The preliminary analyses indicated that no significant differences were detected when sex was included as a covariate. The data of male and female participants were subsequently pooled within each group for the primary analyses. Two-way repeated-measures ANOVA was then employed to explore the independent main effects of group (HIIT2850m, HIIT1220m, CG2850m), time (pretest, posttest), and the group × time interaction effects.

Statistical inference regarding changes was based primarily on the time × group interaction effect**.** When significant group × time interaction effects were observed, post hoc between-group comparisons were performed via independent-samples t tests with Bonferroni adjustment to avoid false positives in multiple comparisons (see Supplementary Tables S1 and S2). To reduce inflation of the adjusted value, we opted to control three post-tests comparisons, resulting in an adjusted significance level of 0.0167. Nevertheless, the study was not statistically powered to detect sex-related interaction effects, and an additional exploratory three-way ANOVA (group × sex × time) analysis was also conducted to explore potential sex interaction effects (see Supplementary Table S3). The statistical significance was set at an a priori α level of 0.05 via SPSS (version 27; IBM Corp., Armonk, NY, USA). To detect the real effect of the intervention, all analyses calculated each test's significance determined by effect size, and partial eta-squared for repeated measures was considered small (0.01–0.05), moderate (0.06–0.13) or large (>0.14), and Cohen's d for the *t*-test was considered (negligible, <0.2; small, 0.2–0.49; moderate, 0.5– 0.79; and large, ≥0.8).^37^

Although baseline characteristics such as age and VO_2_ max may influence the outcomes, adjusting methods for analyses were not employed to reduce model overfitting and maintain power. Instead, participants were stratified and distributed to ensure balanced groups at baseline. In addition, the statistical design employed inherently accounted for variabilities between individuals via within-subject changes over time.

## 3 Results

### 3.1 Baseline characteristics

At baseline, there were no significant differences (p > 0.05) between the groups in terms of anthropometric, training or performance characteristics, as shown in Table 2. On the basis of the reported data, there were no significant differences in training load among the intervention (HIIT2850m, 58.8 ± 5.1 km.wk^−1^; HIIT1220m, 59.1 ± 5 km.wk^−1^) and control group (CG2850m, 58.5 ± 5.2 km.wk^−1^). The usual training loads consisted of a relatively equivalent weekly volume (58 ± 5.1 km.wk^−1^), 60--85 min per session, and an intensity distribution of low-intensity (10%), moderate-intensity (80%) and high-intensity (10%) training, which included five sessions.wk^−1^.

### 3.2 Performance time for the 5000-m race

The baseline 5000-m time was not significantly different among the groups (p = 0.99). Following the intervention, a significant main effect of time (F(1, 39) = 15.13, p = 0.000, η_p_^2^ = 0.28) was found (Table 4), indicating improvement across the intervention period. In contrast, there was no significant difference in the main effect of group (F(2, 39) = 0.12, p = 0.89, η_p_^2^ = 0.006), nor was a significant group by time interaction (F(2,39) = 1.15, p = 0.27, η_p_^2^ = 0.07) detected in 5000 m time. The change in 5000-m time decreased in both intervention groups, with a greater mean reduction (improvements) observed in the HIIT1220m group (Δ: 20.1 ± 23.7 s) than in the HIIT2850m group (Δ: 16.7 ± 25.9 s), whereas the control group (Δ: 6.0 ± 22.9) showed minimal change (Table 3). The findings indicate that running performance improved following the HIIT interventions, although the degree of improvement did not differ significantly among the groups (F(2, 39) = 0.12, p = 0.89, η_p_^2^ = 0.006), with a greater percentage change found in the HIIT2850m (1.4%) and HIIT1220m (1.8%) groups.

**Table 3:** Descriptive statistics and paired samples t tests of 5000 m pacing, and overall time following HIIT at low and moderate altitudes.

Parameter		HIIT1220m (n = 14)			HIIT2850m (n = 14)			CG2850m (n = 14)		
Pace/speed		pre	post	Δ (r)	pre	post	Δ (r)	pre	post	Δ (r)
1^st^ km	min.km^−1^	3.4 ± 0.2	3 ± 0.2	0.4 ± 0.1 (0.96)∗	3.4 ± 0.4	3.2 ± 0.4	0.2 ± 0.1 (0.99)∗	3.4 ± 0.3	3.6 ± 0.3	−0.2 ± 0.1 (0.95)
	km.h^−1^	17.8 ± 1.2	20.2 ± 1.5	−2.4 ± 0.5(0.95)∗	17.9 ± 1.9	19 ± 2	−1.1 ± 0.3 (0.99)∗	17.8 ± 1.3	16.8 ± 1.3	0.97 ± 0.4 (0.94)
2^nd^ km	min.km^−1^	3.5 ± 0.3	3.6 ± 0.2	−0.1 ± 0.1 (0.95)	3.5 ± 0.4	3.6 ± 0.4	−0.03 ± 0.1(0.97)	3.5 ± 0.3	3.3 ± 0.3	0.1 ± 0.1 (0.92)
	km.h^−1^	17.5 ± 1.2	16.8 ± 1.1	0.7 ± 0.4 (0.95)	17.2 ± 1.9	17 ± 1.7	−0.2 ± 0.5 (0.97)	17.3 ± 1.5	18.1 ± 1.7	−0.8 ± 0.6 (0.93)
3^rd^ km	min.km^−1^	3.7 ± 0.3	3.9 ± 0.2	−0.2 ± 0.1 (0.94)∗	3.6 ± 0.5	3.8 ± 0.4	−0.2 ± 0.1 (0.98)	3.6 ± 0.4	3.5 ± 0.4	0.2 ± 0.1(0.98)
	km.h^−1^	16.5 ± 1.1	15.6 ± 0.9	0.9 ± 0.4 (0.93)∗	16.7 ± 2.1	15.9 ± 1.7	0.9 ± 0.5 (0.98)	16.6 ± 1.6	17.4 ± 1.8	−0.7 ± 0.4 (0.98)
4^th^ km	min.km^−1^	3.8 ± 0.3	3.9 ± 0.3	−0.2 ± 0.1 (0.92)∗	3.8 ± 0.5	3.9 ± 0.5	−0.2 ± 0.1(0.97)	3.8 ± 0.4	3.6 ± 0.4	0.2 ± 0.1 (0.97)
	km.h^−1^	16 ± 1	15.3 ± 1	0.7 ± 0.5 (0.91)∗	16.2 ± 2	15.4 ± 1.8	0.8 ± 0.6 (0.97)	16.1 ± 1.8	17 ± 2.1	−0.9 ± 0.5 (0.97)
5^th^ km	min.km^−1^	3.6 ± 0.2	3.2 ± 0.2	0.4 ± 0.1 (0.89)∗	3.9 ± 0.5	3.4 ± 0.4	0.5 ± 0.1(0.97)∗	3.7 ± 0.4	3.9 ± 0.4	−0.2 ± 0.1 (0.97)
	km.h^−1^	16.5 ± 1.1	18.8 ± 1.4	2.3 ± 0.7 (0.87)∗	15.7 ± 2.2	18 ± 2.4	2.3 ± 0.5 (0.98)∗	16.4 ± 1.9	15.6 ± 1.8	−0.8 ± 0.5 (0.96)
Overall	min	17.9 ± 1.2	17.6 ± 1.2	0.3 ± 0.4 (0.95)	18.2 ± 2.2	17.9 ± 2	0.3 ± 0.4 (0.98)	18 ± 1.7	17.9 ± 1.8	0.1 ± 0.4 (0.98)
	min.km^−1^	3.6 ± 0.3	3.5 ± 0.2	0.07 ± 0.08(0.95)	3.6 ± 0.4	3.6 ± 0.4	0.06 ± 0.09(0.98	3.6 ± 0.3	3.6 ± 0.4	0.02 ± 0.07(0.98
	km.h^−1^	16.8 ± 1.1	17.1 ± 1.1	−0.3 ± 0.4 (0.94)	16.7 ± 2	16.9 ± 1.9	−0.2 ± 0.4 (0.98)	16.8 ± 1.6	16.9 ± 1.7	−0.1 ± 0.4(0.98)

***Note***: Pretest and posttest values are presented descriptively. Statistical inference regarding changes over time was based on the group × time interaction from the repeated-measures ANOVA (see Table 4). Bonferroni post hoc correction confirmed that the HIIT1220m group was significantly different at 0.0167, with ∗ CG2850m. **Abbreviations**: min, minutes; min.km^−1^, minutes per kilometer; km.h^−1^, kilometers per hour; n, number of participants; HIIT1220m, high-intensity interval training group at low altitude; HIIT2850m, high-intensity interval training group at moderate altitude; CG2850m, control group at moderate altitude; Δ, paired mean change = posttest-pretest; r, pre-post correlations; km, kilometer.

**Table 4:** Omnibus effects following HIIT at low and moderate altitudes.

Distance	parameter	F value, p value (η_p_^2^)		
		Time	Group	Time x Group
1^st^ km	Pace (min.km^−1^)	139.82, <0.001∗ (0.78)	3.68, 0.034∗ (0.16)	238.18, <0.001∗ (0.92)
	Speed (km.h^−1^)	165.24, <0.001∗ (0.81)	4.13, 0.024∗ (0.18)	224.95, <0.001∗ (0.92)
2^nd^ km	Pace (min.km^−1^)	0.16, 0.693 (0.004)	0.67, 0.518 (0.03)	25.9, <0.001∗ (0.57)
	Speed (km.h^−1^)	0.17, 0.687 (0.00)	0.71, 0.500 (0.035)	27.39, <0.001∗ (0.58)
3^rd^ km	Pace (min.km^−1^)	37.98, <0.001∗ (0.49)	1.24, 0.302 (0.06)	73.89, <0.001∗ (0.79)
	Speed (km.h^−1^)	26.08, <0.001∗ (0.40)	1.37, 0.267 (0.07)	61.35, <0.001∗ (0.759)
4^th^ km	Pace (min.km^−1^)	13.88, <0.001∗ (0.26)	1.17, 0.322 (0.06)	60.33, <0.001∗ (0.76)
	Speed (km.h^−1^)	7.31, 0.01∗ (0.16)	1.32, 0.278 (0.06)	46.35, <0.001∗ (0.7)
5^th^ km	Pace (min.km^−1^)	180.48, <0.001∗ (0.82)	3.38, 0.044∗ (0.15)	135.1, <0.001∗ (0.87)
	Speed (km.h^−1^)	208.84, <0.001∗ (0.84)	3.12, 0.055 (0.14)	133.33, <0.001∗ (0.87)
Overall time	min.	15.13, <0.001∗ (0.28)	0.12, 0.892 (0.006)	1.35, 0.272 (0.07)
	Pace (min.km^−1^)	15.13, <0.001∗ (0.28)	0.12, 0.892 (0.006)	1.35, 0.272 (0.07)
	Speed (km.h^−1^)	14.38, <0.001∗ (0.27)	0.03, 0.97 (0.002)	1.11, 0.34 (0.05)

Abbreviations: η_p_^2^, partial Etta squared; p value, probability value; ∗ indicates significance at p < 0.05.

**Note.** ∗Significance was evaluated via Bonferroni-adjusted *p value < 0.0167.*

### 3.3 Pacing strategy and pacing speed for the 5000 m race

**Fig. 2:**
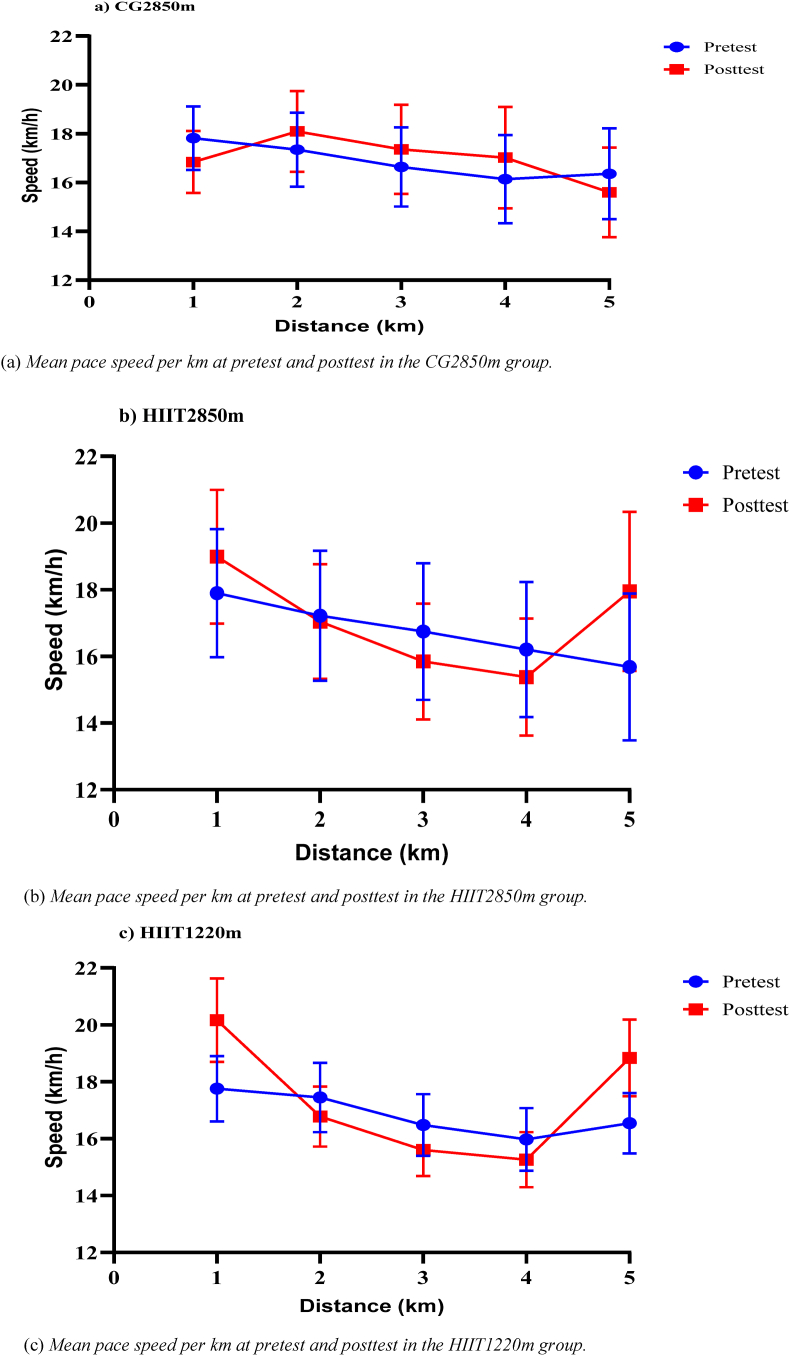
Competitive 5000 m race pacing speed at each km before and after eight weeks of HIIT at two different altitudes: (a) CG2850m, a control group at moderate altitude; (b) HIIT2850m, a high-intensity interval training group at moderate altitude; (c) HIIT1220m, a high-intensity interval training group at low altitude.

The participants used a parabolic reversed J-shaped pacing strategy within groups, as presented in the summary of pacing speed illustrated in Fig. 2 (a, b, and c). These events occurred as a fast starting at the 1^st^ km, followed by a gradual speed reduction until the 4^th^ km, and then a gradual velocity increase to the last (5^th^) km. In particular, the 5^th^ km speed significantly improved in both intervention groups, which clearly indicates a reversed J-shaped pacing strategy.

On the other hand, significant group × time interactions were identified for running speed (km.h^−1^) across all km segments, except for the overall 5000-m running speed (km.h^−1^) (F(2, 39) = 1.11, p = 0.34, η_p_^2^ = 0.05) (Table 4). In addition, a significant main effect of time was observed for speed (km.h^−1^), except at the 2^nd^ km (F(1, 39) = 0.17, p = 0.69, η_p_^2^ = 0.004), whereas the main effect of group revealed significant differences only at the 1^st^ km (F(2, 39.) = 4.13, p = 0.024, η_p_^2^ = 0.18). Descriptively, speed increased in both HIIT groups, with the greatest improvements observed in the HIIT1220m group during the later race segments (Fig. 2). The CG2850m group showed inconsistent changes across the race (Table 3). The post hoc analyses revealed that the HIIT1220m group achieved significantly higher speeds (km.h^−1^) in all km segments, except at the 2^nd^ km segment (t (26) = 2.50, p = 0.019, Cohen's d = 0.94), and the HIIT2850m group presented higher speeds (km.h^−1^) at the 1^st^ (t (26) = 3.39, p = 0.002, Cohen's d = 1.28) and 5^th^ km (t (26) = 2.93, p = 0.007, Cohen's d = 1.11) kilometer segments than did the CG2850m group; however, no significant differences were detected between the HIIT1220m and HIIT2850m groups (Table S1).

### 3.4 Perceived effort of 5000 m running

At baseline, no significant differences were observed among the groups. Following the intervention, participants’ RPE (6–20 scale) responses during the 5000 m race demonstrated significant main effects of time (p < 0.05) across all kilometer segments, indicating overall changes in RPE from pretest to posttest as shown in Table 5. In addition, there was a significant group × time interaction at all km segments, except at the 2^nd^ km (F (2, 39) = 0.14, p = 0.866, η_p_^2^ = 0.007). The analysis also revealed a significant main effects of group at the 1^st^ km (F (2, 39) = 9.93, p < 0.001, η_p_^2^ = 0.34), 4^th^ km (F (2, 39) = 18.89, p < 0.001, η_p_^2^ = 0.49), and 5^th^ km (F (2, 39) = 11.18, p < 0.001, η_p_^2^ = 0.36).

**Table 5:** The perceived feelings reported by the participants via the Borges (6--20) scale at each km during the 5000 m race before and after the HIIT intervention at low and moderate altitudes.

Distance	HIIT1220m (n = 14)		HIIT2850m (n = 14)		CG2850m (n = 14)		F value, p value (η_p_^2^)		
	pre	post	pre	post	pre	post	Time	Group	Time x Group
1^st^ km	13.7 ± 0.7	11.7 ± 0.8∗	13.9 ± 0.9	12.1 ± 0.5∗	13.7 ± 0.9	13.8 ± 0.6	93.66, <0.001∗ (0.71)	9.93, <0.001∗(0.34)	26.68, <0.001∗ (0.58)
2^nd^ km	15.6 ± 0.8	14.9 ± 1.1	15.4 ± 0.8	14.9 ± 1	15.4 ± 0.8	14.6 ± 0.8	16.18, <0.001∗ (0.29)	0.64, 0.534(0.03)	0.14, 0.866 (0.007)
3^rd^ km	16.2 ± 0.9	16 ± 0.7	15.4 ± 0.6	16.1 ± 0.5	15.9 ± 0.7	16.5 ± 0.7	7.81, 0.008∗ (0.17)	3.33, 0.046 (0.15)	4.79, 0.014∗ (0.19)
4^th^ km	17.6 ± 0.5	17.5 ± 0.7∗	16.6 ± 0.5	17.4 ± 0.7∗	17.6 ± 0.5	18.6 ± 0.6	39.63, <0.001∗ (0.5)	18.89, <0.001∗ (0.49)	12.62, <0.001∗ (0.39)
5^th^ km	19.1 ± 1.1	17.2 ± 0.7∗†	19.1 ± 0.5	18.5 ± 0.5∗	19.1 ± 0.7	19.2 ± 0.7	30.98, <0.001∗ (0.44)	11.18, <0.001∗, (0.36)	16.98, <0.001∗ (0.47)

**Note**: Bonferroni post hoc correction confirmed that the HIIT1220m group was significantly different at 0.0167, with ∗ CG2850m, † HIIT2850m; RPE, rated perceived exertion; km, kilometer.

**Fig. 3:**
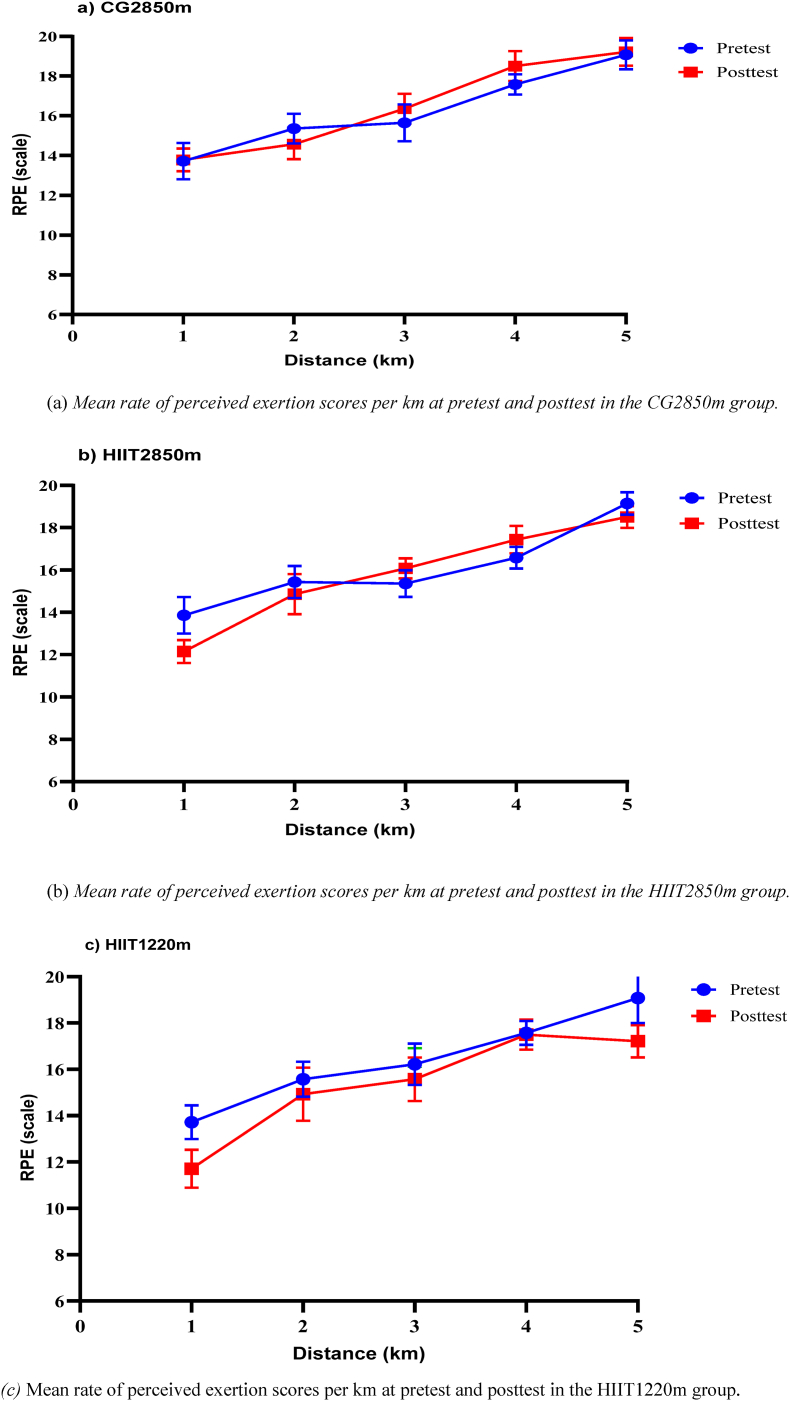
illustrates the perceived feelings during competitive 5000 m race at each km RPE before and after eight weeks of HIIT at two different altitudes: (a) HIIT2850m, (b) CG2850m, and (c) HIIT1220m.

The RPE was reduced in both HIIT groups, with a more pronounced reduction in the HIIT1220 m group, particularly in the later stages of the race as presented in Fig. 3 (a and b and c). The control group exhibited minimal perceptual change (Table 5). However, the post hoc comparisons demonstrated that, compared with the control group, the HIIT2850m and HIIT1220m groups reported significantly lower RPE values in the 1^st^ (t (26) = 7.80, p < 0.001, Cohen's d = 2.95); (t (26) = 7.69, p < 0.001, Cohen's d = 2.91), 4^th^ (t (26) = 5.02, p < 0.001, Cohen's d = 1.89); (t (26) = 4.71, p < 0.001, Cohen's d = 1.78), and 5^th^ (t (26) = 3.07, p = 0.005, Cohen's d = 1.16); (t (26) = 7.57, p < 0.001, Cohen's d = 2.86) kilometers. In contrast, a significant differences were observed between the intervention groups at the 5^th^ km, with the HIIT1220m group reported lower RPE values than did the HIIT2850m group (t (26) = 5.53, p < 0.001, Cohen's d = 2.09) (Table S2).

### 3.5 Sensitivity analyses

Exploratory analysis often involves multiple statistical tests used to test the robustness of results, which increases the risk of false positives due to data-driven searches for significance.^38^ In addition, adjusted multiple testing applications may reduce the statistical power for a given sample size, potentially increasing the likelihood of Type II errors. Taking this into account, we performed an additional sensitivity analysis to address potential sex-related differences by including sex as a factor in three-way repeated-measures ANOVA (group × time × sex) for each kilometer pacing speed and RPE, which is presented in Table S3. The result revealed that men and women differed in overall performance (p < 0.001), whereas no significant sex-related interaction (p > 0.05: sex × time, sex × group, and group × time × sex) effects were found.

On the other hand, G∗Power sensitivity analysis for within–between interactions in the repeated-measures ANOVA was performed for the primary outcome via the actual sample size (n = 42) and 80% power, α = 0.05, indicating that the minimum detectable group × time interaction effect was f = 0.25. This suggests that the observed 5000-m time effect size (f = 0.27, partial η^2^ = 0.07) is above the threshold (f ≥ 0.25) which is sufficient to detect the observed effect. In addition, the achieved power (86%) confirmed that the study was adequately powered to detect reported interaction differences above the minimum power threshold ≥80%. Although the priori calculation was not powered for the secondary outcomes, post hoc sensitivity analysis for RPE indicated that the study was adequately powered (99--100%) to detect an observed large effect (η_p_^2^ = 0.19--0.58, Cohen's f = 0.48-1.18) which is above the effect size threshold (f ≥ 0.25). Likewise, analysis of pacing speed demonstrated a similar robust detection of results as RPE.

## 4 Discussion

Within the current body of evidence, this study is among the first to compare the effects of HIIT performed at low and moderate altitudes on 5000-m performance and the perceived exertion of moderate-altitude resident long-distance runners. The main findings support the hypothesis that an 8-week HIIT intervention led to improvements in 5000-m time, RPE, and pacing speed regardless of the training altitude venue, with greater changes observed in HIIT at low-altitudes.

Pacing strategies are learned behaviors developed through chronic training. In our context, the strategy used to regulate effort and economically distribute energy in the 5000 m running was similar within groups, and was characterized by a reversed J-shaped strategy.

Among the different pacing strategies,^27^ the parabolic shape is more abundant and is associated with the best performance in terms of endurance. In particular, a greater speed is generated at the beginning and end of the race.^27^^,^^39^ Our study confirmed that the pacing strategy remained remarkably consistent across groups while pacing selection is influenced by multiple interacting factors.^29^^,^^40^^,^^41^ This may be attributable to the learned regulation of RPE, which helps to make logical decisions to monitor effort and distribute energy across the race. Previous findings reported a maintained pacing strategy after 4 weeks of HIIT.^25^

This mechanism may translate into improved pacing regulation via internal and external psychophysiology and a reduced perception of effort. This aligns with contemporary evidence showing that HIIT, when appropriately integrated, plays a central role in enhancing endurance performance and pacing regulation.^6^^,^^8^^,^^42^ In our study, the subjective feeling of perceived effort was reduced in both intervention groups. Reductions in RPE, reflected by moderate to large effect sizes, indicate improved perceptual regulation, enabling athletes to sustain higher speeds with lower subjective effort. While the RPE is influenced by several factors, it monitors the effort and balanced distribution of energy to the last laps of a race distance. This finding concurs with the result of Silva and colleagues,^25^ who reported similar RPE values following 4 weeks of HIIT. This may be due to the short HIIT period and limited recording point.

According to our results, HIIT intervention greatly influences pacing speed, particularly at the first and last kilometer paces. Notably, participants who completed the race at low altitudes tended to show greater improvements in speed across each kilometer segment of the race, except at the 2^nd^ km, than did the control group. These early and late kilometer paces are crucial in making pacing decisions and fatigue tolerance, which suggests that HIIT plays a potential role in facilitating competition efforts. The possible observed changes may be related to the standardized nature of the intervention, which consisted of structured work and recovery intervals. The large to very large effect sizes observed in the group × time interactions reinforce that these improvements extend beyond statistical significance and are of clear practical relevance for competitive performance. Each interval monitored pace, and perceived exertion remains crucial to transfer for race performance adaptability.^43^ The work distribution across intervals also influences motor unit recruitment and metabolic systems to develop 5 km pacing skills and physiological quality.^44^

In addition, HIIT at lower altitudes likely enables athletes to sustain greater absolute intensities, which contributes to the adoption of high running speeds by athletes. This may have increased the effective pacing speed across the distance. These findings indicate that improved aerobic and anaerobic capacity following HIIT improves pacing speed and endurance performance.^45^ In addition, an improvement in the energy cost of running and ground contact time after HIIT contributes to altering pacing speed and improving race performance (Marmondi et al., 2025). The improved muscle efficiency, enhanced buffering capacity, and neuromuscular coordination improve tolerance to high-intensity running and support more stable pacing, particularly during the later stages of a race.^7^^,^^46^

These effective pacing speeds potentially contributed to a 5000 m time improvement to a similar degree, with greater improvement in the intervention groups. Although the group × time interaction effect did not reach statistical significance, the moderate effect size and consistent improvements suggest that the intervention led to meaningful improvements in 5000 m performance. A possible explanation for the same rate across time is that our study participants maintained the usual training, which helped with significant progress in all the groups. In contrast, the addition of high-quality training contributes to increasing the rate of performance gains.^45^^,^^47^ A time improvement of 5000 m for HIIT at moderate (1.4%) and low (1.8%) altitudes was observed in the present study. This aligns with the findings of Park et al.,^20^ who reported 3 km time improvements after 6 weeks of HIIT under hypoxia (4.9%) and normoxia (3.4%). In addition, Silva et al.^25^ evaluated a 4-week HIIT effect on a 5-km trial and reported an approximately 2.5% improvement in performance time. Robertson et al.^48^ also reported a 1.1% improvement after LHTL-H, and other studies after the LHTL protocol reported improvements in endurance performance.^23^^,^^49^^,^^50^

The underlying reason might be that sustained training speed and oxygen availability during HIIT possibly influenced performance compared with the limited VO_2_ max during hypoxia and the less stressed LLTL protocol. This aligns with contemporary evidence showing that HIIT, when appropriately integrated, plays a central role in enhancing endurance performance and pacing regulation.^6^^,^^8^^,^^42^ Importantly, these outcomes may be attributed to enhanced physiological efficiency and improved pacing regulation developed through repeated HIIT enabling runners to sustain higher speeds while experiencing effort as more manageable (Marmondi et al., 2025).

Although logistical issues in the natural LHTL have guaranteed benefits for performance, simulated altitudes are less effective and generate conflicting results.^17^ In contrast, our findings concur with insignificant results reported following HIIT under hypoxia and normoxia for 3 km time.^21^ There was also no change in 5 km time for either the LHTL or LHTL groups.^51^ These contradictory results may be due to study design, altitude type/protocol, and interindividual variations among studies.

### 4.1 Limitations and future directions

There are several limitations that should be acknowledged when the findings are interpreted and considered in future studies. First, the sample compositions (male and female participants) were merged without appropriate statistical control, and pooled within each group for analysis because of the relatively small sample size, which limited the ability to examine sex-related responses. Although exploratory analyses including sex as a factor and cofactor were conducted, the study was not sufficiently powered to detect potential sex-based interactions. This may offer insights that could be used to test hypothesis in the future. As expected, men and women differed in overall performance; however, no significant sex-related interactions were observed. These findings indicate that while absolute performance varies by sex, the relative adaptation pattern was comparable to that of the intervention across sexes. Accordingly, the findings should be interpreted with caution. In addition, the study was not powered a priori for pacing and perceptual outcomes, although sensitivity analyses suggest adequate detection of medium effects. Future research should employ a priori power calculations, larger and more balanced sample sizes, and, where feasible, crossover study designs to allow a more robust examination of sex differences that may influence the outcomes.

Another limitation related to the study population was that moderate-altitude resident distance runners and differences in residency and training characteristics compared with lower-altitude athletes may limit the generalizability of the findings across different altitude resident athletes. Moreover, while running competitions are typically actual group races, we examined the study variables in the presence of other runners which are dominant over individual pacing.^52^ This approach may have constrained individual variability in the pacing strategy. Self-selected pacing strategies and tactical positioning (leading, chasing, pack and overall running) may provide a more comprehensive picture of performance. Furthermore, while we managed to create equivalent groups at baseline, considering covariates such as training and competition experience, as well as physiological and performance characteristics, may have contributed to the intersubject variability in the intervention responses. Consequently, determining individual dose‒response relationships remains an important area for further research.

Regarding measurement considerations, training intensity (vVo_2_ max) was determined indirectly via a 5-min maximal aerobic speed test. While this method is reliable and valid, we acknowledge that it may have been sensitive enough to detect relative performance. More rigorous direct laboratory outcomes are therefore needed to determine absolute performance. In addition, the RPE represents a valid, widely used, and easily accessible tool to determine training intensity and pacing regulation.^53^^,^^54^ However, as a subjective measure, it may be influenced by participants' expectations,^55^ as well as intrinsic and extrinsic psychophysiological factors known to affect pacing and feelings of exertion.^56^ To mitigate these biases, the participants were repeatedly reminded that there were no correct or incorrect answers and were encouraged to report their perceptions as honestly as possible. The use of kilometer split times and gross pacing analysis may not genuinely indicate the detailed work distribution across 5000 m performance. Specifically, pacing analysis using shorter distance segments (e.g., 100 m and 400 m) may better characterize the pacing strategy. Despite careful control of before, during and after travel to low altitude, as well as standardized recovery, the present study did not include direct assessments of fatigue and recovery status. Unquantified individual differences may have influenced the observed outcomes. In addition, while training load protocols were standardized, altitude-related variations may have led to differences in internal physiological load, running cost, and relative exercise intensity, which was central to the study's purpose. For better quality, incorporating internal load measures such as heart rate, lactate concentration, and ventilation rate would enable better characterization of individual responses.

Finally, the pacing strategy is influenced by multiple contextual factors inherent to outdoor field testing, including wind conditions, terrain, and environmental variability. Although the measurements were conducted under similar conditions, residual environmental effects cannot be fully excluded. Future studies may benefit from conducting measurements in more controlled indoor environments. While short-term HIIT interventions have consistently improved endurance performance, more differentiated adaptations may require longer durations to intensify training stimuli.^6^ Accordingly, future research should extend the training duration and systematically manipulate the interval structure, intensity distribution, work-to-rest ratio, pacing strategies and environmental exposure of several HIIT modalities to better characterize the magnitude and persistence of adaptations.

## 5 Conclusion

Our study presents evidence that HIIT at low and moderate altitudes is beneficial for improving pacing speed and RPE, which in turn improved 5000 m performance time. In particular, the findings underscore the practical advantage of HIIT at low altitudes in shaping race pacing and perceived effort which generated greater significant improvements in the 1^st^ and 5^th^ km paces, as well as in the 5000 m race time and RPE. This suggests that this high-intensity quality training is effective in optimizing performance. This finding also strengthens the view that performing HIIT at low altitudes can be a strategic complement to moderate-altitude resident athletes for greater performance gains. In light of these findings, it is recommended that moderate-altitude endurance athletes and coaches incorporate high-intensity training in their plans at lower altitudes to promote performance. This would be effective in event-specific weekly (microcycle) sessions of an undulating step loading macrocyle, specifically during a specific preparation phase of the training program.

## Authors' contributions

SF, TT, and ZB contributed to the study design and conceptualization. SF, TT, and ZB were involved in data curation and formal analysis. SF drafted the manuscript. TT was responsible for randomization, supervision, and validation. TT and ZB provided review and editing. All authors contributed to the manuscript process and approved the final version.

## Ethical statement

The study protocol was carried out according to the Helsinki Declaration. The experimental procedures were submitted, and the Sport Academy ethical committee at Bahir Dar University approved the study [App. No: IRERC 06/2024]. A written informed consent was obtained from each participant following a detailed verbal and written explanation of the study risks, benefits and procedure before taking part.

## Availability of data and materials

The datasets used and/or analysed during the current study are available from the corresponding author on reasonable request.

## Funding

Debark University under the Ethiopia ministry of education fully funded this work as a PhD research project.

## Declarations of interest statement

The authors declare that they have no competing interests.

## References

[1] Falk Neto J.H., Faulhaber M., Kennedy M.D. (2024). The characteristics of endurance events with a variable pacing profile-time to embrace the concept of "intermittent endurance events"?. Sports (Basel).

[2] Haddad M., Chaouachi A., Wong del P. (2014). Influence of exercise intensity and duration on perceived exertion in adolescent Taekwondo athletes. Eur J Sport Sci.

[3] Bonato G., Goodman S.P.J., Tjh L. (2023). Physiological and performance effects of live high train low altitude training for elite endurance athletes: a narrative review. Curr Res Physiol.

[4] Fentaw S., Tadesse T., Birhanu Z. (2025). Methodological and aerobic capacity adaptations of high‐intensity interval training at different altitudes in distance runners: a comprehensive meta‐analysis. Physiological Reports.

[5] Atakan M.M., Li Y., Koşar Ş.N., Turnagöl H.H., Yan X. (2021). Evidence-Based effects of high-intensity interval training on exercise capacity and health: a review with historical perspective. Int J Environ Res Publ Health.

[6] Buchheit M., Laursen P.B. (2013). High-intensity interval training, solutions to the programming puzzle: part I: cardiopulmonary emphasis. Sports Med.

[7] MacInnis M.J., Gibala M.J. (2017). Physiological adaptations to interval training and the role of exercise intensity. The Journal of physiology.

[8] Casado A., González-Mohíno F., González-Ravé J.M., Foster C. (2022). Training periodization, methods, intensity distribution, and volume in highly trained and elite distance runners: a systematic review. Int J Sports Physiol Perform.

[9] Seiler K.S., Kjerland G. (2006). Quantifying training intensity distribution in elite endurance athletes: is there evidence for an "optimal" distribution?. Scand J Med Sci Sports.

[10] Hov H., Wang E., Lim Y.R. (2023). Aerobic high-intensity intervals are superior to improve VO(2max) compared with sprint intervals in well-trained men. Scand J Med Sci Sports.

[11] Li F., Kong Z., Zhu X. (2022). High-intensity interval training elicits more enjoyment and positive affective valence than moderate-intensity training over a 12-week intervention in overweight young women. Journal of Exercise Science & Fitness.

[12] Marmondi, F., Panascì, M., Filipas, L., Faelli, E. L., & Bonato, M. High-Intensity interval vs moderate-intensity continuous training in endurance runners: a systematic review of physiological, biochemical, physical, and biomechanical adaptations. Int J Sports Sci Coach, 0(0), 17479541251375315. 10.1177/17479541251375315.

[13] Saunders P.U., Telford R.D., Pyne D.B., Gore C.J., Hahn A.G. (2009). Improved race performance in elite middle-distance runners after cumulative altitude exposure. Int J Sports Physiol Perform.

[14] Stray-Gundersen J., Levine B.D. (2008). Live high, train low at natural altitude. Scand J Med Sci Sports.

[15] Clark S.A., Bourdon P.C., Schmidt W. (2007). The effect of acute simulated moderate altitude on power, performance and pacing strategies in well-trained cyclists. Eur J Appl Physiol.

[16] Koehle M.S., Cheng I., Sporer B. (2014). Canadian Academy of Sport and Exercise Medicine position statement: athletes at high altitude. Clin J Sport Med.

[17] Bonetti D.L., Hopkins W.G. (2009). Sea-level exercise performance following adaptation to hypoxia: a meta-analysis. Sports Med.

[18] Hamlin M.J., Lizamore C.A., Hopkins W.G. (2018). The effect of natural or simulated altitude training on high-intensity intermittent running performance in team-sport athletes: a meta-analysis. Sports Med.

[19] Kong Z., Lei O.K., Sun S. (2022). Hypoxic repeated sprint interval training improves cardiorespiratory fitness in sedentary young women. Journal of Exercise Science & Fitness.

[20] Park H.Y., Jung W.S., Kim S.W., Kim J., Lim K. (2022). Effects of interval training under hypoxia on hematological parameters, hemodynamic function, and endurance exercise performance in amateur female runners in Korea. Front Physiol.

[21] Jung W.S., Kim S.W., Park H.Y. (2020). Interval hypoxic training enhances athletic performance and does not adversely affect immune function in Middle- and long-distance runners. Int J Environ Res Public Health.

[22] Robach P., Bonne T., Flück D. (2014). Hypoxic training: effect on mitochondrial function and aerobic performance in hypoxia. Med Sci Sports Exerc.

[23] Park H.Y., Park W., Lim K. (2019). Living high-training low for 21 days enhances exercise economy, hemodynamic function, and exercise performance of competitive runners. J Sports Sci Med.

[24] Robach P., Hansen J., Pichon A. (2018). Hypobaric live high-train low does not improve aerobic performance more than live low-train low in cross-country skiers. Scand J Med Sci Sports.

[25] Silva R., Damasceno M., Cruz R. (2017). Effects of a 4-week high-intensity interval training on pacing during 5-km running trial. Braz J Med Biol Res.

[26] Bejder J., Nordsborg N.B. (2018). Specificity of "Live High-Train Low" altitude training on exercise performance. Exerc Sport Sci Rev.

[27] Abbiss C.R., Laursen P.B. (2008). Describing and understanding pacing strategies during athletic competition. Sports Med.

[28] St Clair Gibson A., Lambert E.V., Rauch L.H. (2006). The role of information processing between the brain and peripheral physiological systems in pacing and perception of effort. Sports Med.

[29] Sha J., Yi Q., Jiang X., Wang Z., Cao H., Jiang S. (2024). Pacing strategies in marathons: a systematic review. Heliyon.

[30] Liu Y., Xia Y., Yue T. (2023). Adaptations to 4 weeks of high-intensity interval training in healthy adults with different training backgrounds. Eur J Appl Physiol.

[31] Panascì M., Castagna C., Rago V., Ferrando V., Ruggeri P., Faelli E. (2025). Effects of a 4-Week Off-Season High-Intensity Training Program on aerobic performance and sprint endurance ability in adolescent female football players: a pilot Study. Journal of Functional Morphology and Kinesiology.

[32] Lenk M., Matzka M., Lauber L., Kunz P., Sperlich B. (2025). Impact of weekly frequency of high-intensity interval training on cardiorespiratory, metabolic, and performance measures in recreational runners - an exploratory study. Physiol Rep.

[33] Berthon P., Fellmann N., Bedu M. (1997). A 5-min running field test as a measurement of maximal aerobic velocity. Eur J Appl Physiol Occup Physiol.

[34] Chamoux A., Berthon P., Laubignat J.F. (1996). Determination of maximum aerobic velocity by a five minute test with reference to running world records. A theoretical approach. Arch Physiol Biochem.

[35] Borg G.A. (1982). Psychophysical bases of perceived exertion. Med Sci Sports Exerc.

[36] Christen J., Foster C., Porcari J.P., Mikat R.P. (2016). Temporal robustness of the session rating of perceived exertion. Int J Sports Physiol Perform.

[37] Cohen J. (2013).

[38] Ditroilo M., Mesquida C., Abt G., Lakens D. (2025). Exploratory research in sport and exercise science: perceptions, challenges, and recommendations. J Sports Sci.

[39] Casado A., Hanley B., Jiménez-Reyes P., Renfree A. (2021). Pacing profiles and tactical behaviors of elite runners. Journal of Sport and Health Science.

[40] Baron B., Moullan F., Deruelle F., Noakes T.D. (2011). The role of emotions on pacing strategies and performance in middle and long duration sport events. Br J Sports Med.

[41] Davies M.J., Clark B., Welvaert M. (2016). Effect of environmental and feedback interventions on pacing profiles in cycling: a meta-analysis. Front Physiol.

[42] Seiler S. (2010). What is best practice for training intensity and duration distribution in endurance athletes?. Int J Sports Physiol Perform.

[43] McClean Z., Iannetta D., Macinnis M., Aboodarda S.J. (2023). Shorter high-intensity cycling intervals reduce performance and perceived fatigability at work-matched but not task failure. Med Sci Sports Exerc.

[44] Zadow E.K., Gordon N., Abbiss C.R., Peiffer J.J. (2015). Pacing, the missing piece of the puzzle to high-intensity interval training. Int J Sports Med.

[45] Liu Y., Abdullah B.B., Abu Saad H.B. (2024). Effects of high-intensity interval training on strength, speed, and endurance performance among racket sports players: a systematic review. PLoS One.

[46] Hung C.H., Su C.H., Wang D. (2025). The role of high-intensity interval training (HIIT) in neuromuscular adaptations: implications for strength and power Development-A review. Life.

[47] Reuter M., Rosenberger F., Barz A. (2023). Does higher intensity increase the rate of responders to endurance training when total energy expenditure remains constant? A randomized controlled trial. Sports Med Open.

[48] Robertson E.Y., Saunders P.U., Pyne D.B., Gore C.J., Anson J.M. (2010). Effectiveness of intermittent training in hypoxia combined with live high/train low. Eur J Appl Physiol.

[49] Garvican L.A., Pottgiesser T., Martin D.T., Schumacher Y.O., Barras M., Gore C.J. (2011). The contribution of haemoglobin mass to increases in cycling performance induced by simulated LHTL. Eur J Appl Physiol.

[50] Sinex J.A., Chapman R.F. (2015). Hypoxic training methods for improving endurance exercise performance. Journal of Sport and Health Science.

[51] Yi L., Wu J., Yan B. (2024). Effects of three weeks base training at moderate simulated altitude with or without hypoxic residence on exercise capacity and physiological adaptations in well-trained male runners. PeerJ.

[52] Tomazini F., Pasqua L.A., Damasceno M.V. (2015). Head-to-head running race simulation alters pacing strategy, performance, and mood state. Physiol Behav.

[53] Alves D.L., Cruz R., Bara C.L., Osiecki R., Lima J.R., De-Oliveira F.R. (2020). Pre-planned vs. executed real-time pacing strategies during 3-km race: role of rating perceived exertion. Res Q Exerc Sport.

[54] Hardy C.J., Rejeski W.J. (1989). Not what, but how one feels: the measurement of affect during exercise. J Sport Exerc Psychol.

[55] Mothes H., Leukel C., Seelig H., Fuchs R. (2017). Do placebo expectations influence perceived exertion during physical exercise?. PLoS One.

[56] Tucker R., Noakes T.D. (2009). The physiological regulation of pacing strategy during exercise: a critical review. Br J Sports Med.

