# Corrigendum to “Comparative effects of high-intensity interval training at low and moderate altitudes on 5,000-m performance and perceptual responses: A randomized controlled trial” [J Exerc Sci Fit 24 (2) (2026) 200460]

**DOI:** 10.1016/j.jesf.2026.200480

**Published:** 2026-05-16

**Authors:** Sisay Fentaw, Tefera Tadesse, Zerihun Birhanu

**Affiliations:** a Sport Academy, Bahir Dar University, Bahir Dar, Ethiopia; b Sport Science Department, Debark University, Debark, Ethiopia; c Educational Development and Quality Center, University of Global Health Equity, Kigali, Rwanda

**The authors regret:**

In the originally published version of this article, the affiliation details were incomplete. The complete and correct affiliation is shown below:

^*a*^ Department of Sport Science, Sport Academy, Bahir Dar University, P.O. Box 79, Bahir Dar, Ethiopia

^b^ Department of Sport Science, College of Natural and Computational Science, Debark University, Debark, Ethiopia

^c^ Educational Development and Quality Center, University of Global Health Equity, Kigali, Rwanda

**The authors would like to apologise for any inconvenience caused.**

