# Supplementary material for: Comparative effects of high-intensity interval training at low and moderate altitudes on 5000-m performance and perceptual responses: A randomized controlled trial
Source: J Exerc Sci Fit. 2026 Feb 18;24(2):200460. doi: 10.1016/j.jesf.2026.200460 (PMC12950428; doi:10.1016/j.jesf.2026.200460)
Supplement: Multimedia component 1 [file mmc1.docx]

**Supplementary**

**Pacing speed**

The post hoc analyses revealed that the **HIIT1220m group achieved significantly higher speeds** (km.h^-1^) **in all km segments, except at the 2^nd^ km segment (t(26) = 2.50, p = 0.019,** Cohen’s d = 0.94**), and** the HIIT2850m group presented higher speeds (km.h^-1^) at the 1^st^ **(t (26) = 3.39, p = 0.002,** Cohen’s d = 1.28**)** and 5^th^ km **(t (26) = 2.93, p = 0.007,** Cohen’s d = 1.11**) kilometer** segments than did the CG2850m group; however, no significant differences were detected between the HIIT1220m and HIIT2850m groups (Table S1).

**Table S1** Between-group differences in each kilometer posttest speed via Bonferroni-corrected post-hoc comparisons.

| **Parameter** | **Comparison** | **MD** | **95% CI** | **t** | **df** | **p value** | **Cohen’s d [CI)** |
| --- | --- | --- | --- | --- | --- | --- | --- |
| 1^st^ km | CG2850m vs HIIT2850m | -2.15±0.6 | [-3.45, -0.85] | -3.39 | 26 | 0.002* | -1.28 [-2.09, -0.45] |
|  | CG2850m vs HIIT1220m | -3.33±0.5 | [-4.39, -2.26] | -6.40 | 26 | <0.001* | -2.43 [-3.4, -1.42] |
|  | HIIT2850m vs HIIT1220m | -1.18±0.7 | [-2.54, 0.19] | -1.78 | 26 | 0.088 | -0.67 [-1.43, 0.09] |
| 2^nd^ km | CG2850m vs HIIT2850m | 1.05±0.6 | [-0.27, 2.36] | 1.64 | 26 | 0.113 | 0.62 [-0.15, 1.37] |
|  | CG2850m vs HIIT1220m | 1.31±0.5 | [0.23, 2.39] | 2.50 | 26 | 0.019 | 0.94 [0.15, 1.72] |
|  | HIIT2850m vs HIIT1220m | 0.27±0.5 | [-0.85, 1.38] | 0.49 | 26 | 0.628 | 0.19 [-0.56, 0.93] |
| 3^rd^ km | CG2850m vs HIIT2850m | 1.51±0.7 | [0.12, 2.89] | 2.24 | 26 | 0.034 | 0.85 [0.06, 1.61] |
|  | CG2850m vs HIIT1220m | 1.74±0.6 | [0.63, 2.87] | 3.20 | 19.18 | 0.004* | 1.21 [0.39, 2.01] |
|  | HIIT2850m vs HIIT1220m | 0.24±0.5 | [-0.85, 1.34] | 0.46 | 19.74 | 0.649 | 0.18 [-0.57, 0.92] |
| 4^th^ km | CG2850m vs HIIT2850m | 1.63±0.7 | [0.14, 3.12] | 2.24 | 26 | 0.034 | 0.85 [0.07, 1.62] |
|  | CG2850m vs HIIT1220m | 1.76±0.6 | [0.47, 3.04] | 2.86 | 18.43 | 0.01* | 1.08 [0.28, 1.87] |
|  | HIIT2850m vs HIIT1220m | 0.13±0.5 | [-0.99, 1.24] | 0.23 | 20.3 | 0.818 | 0.09 [-0.65, 0.83] |
| 5^th^ km | CG2850m vs HIIT2850m | -2.36±0.8 | [-4.01, -0.70] | -2.93 | 26 | 0.007* | -1.11 [-1.89, -0.29] |
|  | CG2850m vs HIIT1220m | -3.25±0.6 | [-4.49, -1.99] | -5.33 | 26 | <0.001* | -2.02 [-2.92, -1.08] |
|  | HIIT2850m vs HIIT1220m | -0.89±0.7 | [-2.41, 0.64] | -1.2 | 2.05 | 0.238 | -0.46 [-1.21, 0.29] |

**Note.** *Significance was evaluated via Bonferroni-adjusted p value < 0.0167.

*Abbreviations: MD,* mean difference ± standard error difference; *CI, confidence interval; df, degree of freedom; t, t statistic; p value, probability value.*

**Perceived exertion**

The post hoc comparisons demonstrated that, compared with the control group, the HIIT2850m and HIIT1220m groups reported significantly lower RPE values in the 1^st^ **(t (26) = 7.80, p < 0.001,** Cohen’s d = 2.95**); (t (26) = 7.69, p < 0.001,** Cohen’s d = 2.91**),** 4^th^ **(t (26) = 5.02, p < 0.001,** Cohen’s d = 1.89**); (t (26) = 4.71, p < 0.001,** Cohen’s d = 1.78**),** and 5^th^ **(t (26) = 3.07, p = 0.005,** Cohen’s d = 1.16**); (t (26) = 7.57, p < 0.001,** Cohen’s d = 2.86**)** kilometers. In contrast, a significant difference was observed between the intervention groups at the 5^th^ km, with the HIIT1220m group reported lower RPE values than did the HIIT2850m group **(t (26) = 5.53, p < 0.001,** Cohen’s d = 2.09**)** (Table S2).

**Table S2** Between-group differences in each kilometer posttest RPE via Bonferroni-corrected post-hoc comparisons.

| **Parameter** | **Comparison** | **MD** | **95% CI** | **t** | **df** | **p value** | **Cohen’s d [CI)** |
| --- | --- | --- | --- | --- | --- | --- | --- |
| 1^st^ km | CG2850m vs HIIT2850m | 1.64±0.2 | [1.21, 2.08] | 7.8 | 26 | <0.001* | 2.95 [1.85, 4.02] |
|  | CG2850m vs HIIT1220m | 2.07±0.3 | [1.52, 2.63] | 7.69 | 26 | <0.001* | 2.91 [1.81, 3.97] |
|  | HIIT2850m vs HIIT1220m | 0.43±0.3 | [-0.11, 0.7] | 1.6 | 26 | 0.115 | 0.62 [-0.15, 1.37] |
| 2^nd^ km | CG2850m vs HIIT2850m | -0.29±0.3 | [-0.95, 0.38] | -0.88 | 26 | 0.386 | -0.33 [-1.08, 0.42] |
|  | CG2850m vs HIIT1220m | -0.36±0.37 | [-1.11, 0.39] | -0.98 | 26 | 0.338 | -0.37 [-1.11, 0.38] |
|  | HIIT2850m vs HIIT1220m | -0.07±0.4 | [-0.8, 0.74] | -0.18 | 25.17 | 0.85 | -0.07 [-0.81, 0.67] |
| 3^rd^ km | CG2850m vs HIIT2850m | 0.43±0.2 | [-0.01, 0.87] | 1.99 | 26 | 0.057 | 0.75 [0.02, 1.51] |
|  | CG2850m vs HIIT1220m | 0.5±0.25 | [-0.02, 1.02] | 1.99 | 26 | 0.057 | 0.75 [-0.02, 1.51] |
|  | HIIT2850m vs HIIT1220m | 0.07±0.2 | [-0.38, 0.53] | 0.32 | 26 | 0.75 | 0.12 [-0.62, 0.86] |
| 4^th^ km | CG2850m vs HIIT2850m | 1.21±0.2 | [0.72, 1.71] | 5.02 | 26 | <0.001* | 1.89 [0.98, 2.79] |
|  | CG2850m vs HIIT1220m | 1.14±0.2 | [0.64, 1.64] | 4.71 | 26 | <0.001* | 1.78 [0.89, 2.65] |
|  | HIIT2850m vs HIIT1220m | -0.07±0.3 | [-0.58, 0.43] | -0.29 | 26 | 0.773 | 0.11 [-0.85, 0.63] |
| 5^th^ km | CG2850m vs HIIT2850m | 0.71±0.2 | [0.24, 1.19] | 3.07 | 26 | 0.005* | 1.16 [0.35, 1.96] |
|  | CG2850m vs HIIT1220m | 2±0.3 | [1.46, 2.54] | 7.57 | 26 | <0.001* | 2.86 [1.78, 3.92] |
|  | HIIT2850m vs HIIT1220m | 1.29±0.2 | [0.81, 1.77] | 5.53 | 26 | <0.001* | 2.09 [1.14, 3.01] |

**Note.** *Significance was evaluated via Bonferroni-adjusted p value < 0.0167.

*Abbreviations: MD,* mean difference ± standard error difference; *CI, confidence interval; df, degree of freedom; t, t statistic; p value, probability value.*

**Exploratory analysis**

**Table S3** Exploratory three-way repeated-measures ANOVA examining the effects of group, time, and sex on each km pace, speed and perceptual response.

| **Distance** | **parameter** | **F value, p value (η_p_²)** | | | | | | |
| --- | --- | --- | --- | --- | --- | --- | --- | --- |
|  |  | **Time** | **Group** | **Sex** | **Time x Group** | **Time x Sex** | **Group x Sex** | **Group x Time x Sex** |
| 1^st^ km | Pace (min.km^-1^) | **129.49, 0.001* (0.78)** | **7.53, 0.002* (0.295)** | **40.33, <0.001* (0.53)** | **217.16, <0.001* (0.92)** | **0.2, 0.66 (0.006)** | **0.74, 0.49 (0.04)** | **0.14, 0.87 (0.008)** |
|  | Speed (km.h^-1^) | **188.4, <0.001* (0.84)** | **8.7, <0.001* (0.32)** | **43.84, <0.001* (0.55)** | **251.47, <0.001* (0.93)** | **2.55, 0.119 (0.06)** | **1.09, 0.348 (0.06)** | **3.71, 0.034 (0.17)** |
|  | **RPE**  **(6-20 scale)** | **90.56, <0.001* (0.72)** | **9.71, <0.001* (0.35)** | **0.15, 0.7 (0.004)** | **27.92, 0.001* (0.61)** | **0.38, 0.001 (0.54)** | **0.59, 0.56 (0.03)** | **1.51, 0.235 (0.08)** |
| 2^nd^ km | Pace (min.km^-1^) | **0.08, 0.79 (0.002)** | **0.96, 0.39 (0.05)** | **30.84, <0.001* (0.46)** | **24.14, <0.001* (0.57)** | **0.55, 0.46 (0.02)** | **0.85, 0.44 (0.05)** | **0.18, 0.84 (0.01)** |
|  | Speed (km.h^-1^) | **0.09, 0.761 (0.00)** | **1.05, 0.361 (0.06)** | **30.57, <0.001* (0.46)** | **24.89, <0.001* (0.58)** | **0.49, 0.488 (0.01)** | **0.75, 0.478 (0.04)** | **0.28, 0.76 (0.02)** |
|  | **RPE**  **(6-20 scale)** | **14.74, <0.001* (0.29)** | **0.96, 0.39 (0.05)** | **0.31, 0.58 (0.009)** | **0.12, 0.89 (0.007)** | **1.01, 0.322 (0.03)** | **2.59, 0.09 (0.13)** | **0.11, 0.89 (0.006)** |
| 3^rd^ km | Pace (min.km^-1^) | **34.28, 0.001* (0.49)** | **1.87, 0.17 (0.09)** | **24.77, 0.001* (0.41)** | **67.31, 0.001* (0.79)** | **0.12, 0.73 (0.003)** | **1.003, 0.38 (0.05)** | **0.09, 0.92 (0.005)** |
|  | Speed (km.h^-1^) | **25.43, <0.001* (0.41)** | **2, 0.149 (0.1)** | **23.52, <0.001* (0.39)** | **58.68, <0.001* (0.77)** | **0.72, 0.402 (0.02)** | **0.83, 0.446 (0.04)** | **1.09, 0.345 (0.06)** |
|  | **RPE**  **(6-20 scale)** | **7.6, 0.009 (0.175)** | **4.12, 0.02* (0.19)** | **4.45, 0.04* (0.11)** | **5.72, 0.007* (0.24)** | **1.64, 0.21 (0.04)** | **1.49, 0.24 (0.08)** | **1.64, 0.21 (0.08)** |
| 4^th^ km | Pace (min.km^-1^) | **13.11, <0.001* (0.27)** | **1.66, 0.2 (0.08)** | **23.91, <0.001* (0.399)** | **54.77, <0.001* (0.75)** | **0.17, 0.68 (0.005)** | **0.75, 0.48 (0.04)** | **0.03, 0.97 (0.002)** |
|  | Speed (km.h^-1^) | **7.52, 0.009* (0.17)** | **1.79, 0.18 (0.09)** | **21.57, <0.001* (0.38)** | **43.54, <0.001* (0.71)** | **0.001, 0.97 (0.00)** | **0.58, 0.57 (0.03)** | **1.22, 0.31 (0.06)** |
|  | **RPE**  **(6-20 scale)** | **42.79, <0.001* (0.54)** | **20.8, <0.001* (0.54)** | **0.09, 0.76 (0.003)** | **15.01, 0.001* (0.46)** | **0.09, 0.77 (0.002)** | **3.57, 0.04 (0.17)** | **3.09, 0.06 (0.15)** |
| 5^th^ km | Pace (min.km^-1^) | **191.6, 0.001* (0.84)** | **5.62, 0.008* (0.24)** | **25.6, <0.001* (0.42)** | **147.6, <0.001* (0.89)** | **0.08, 0.78 (0.002)** | **0.98, 0.38 (0.05)** | **3.38, 0.05 (0.16)** |
|  | Speed (km.h^-1^) | **212.9, <0.001* (0.86)** | **5.16, 0.01* (0.22)** | **26.05, <0.001* (0.42)** | **138.35, <0.001 (0.89)** | **3.29, 0.078 (0.08)** | **0.99, 0.38 (0.05)** | **1.15, 0.327 (0.06)** |
|  | **RPE**  **(6-20 scale)** | **29.56, <0.001* (0.45)** | **9.93, <0.001* (0.36)** | **0.67, 0.42 (0.02)** | **17.47, <0.001* (0.49)** | **0.88, 0.36 (0.02)** | **0.52, 0.59 (0.03)** | **0.86, 0.43 (0.05)** |
| Overall time | Pace (min.km^-1^) | **13.89, <0.001* (0.28)** | **0.09, 0.92 (0.005)** | **29.08, <0.001* (0.45)** | **1.26, 0.297 (0.07)** | **0.11, 0.75 (0.003)** | **0.85, 0.44 (0.05)** | **0.21, 0.81 (0.01)** |
|  | Speed (km.h^-1^) | **12.76, 0.001* (0.26)** | **0.02, 0.98 (0.001)** | **28.86, <0.001* (0.45)** | **1.04, 0.365 (0.05)** | **0.09, 0.76 (0.003)** | **0.86, 0.43 (0.05)** | **0.27, 0.76 (0.02)** |

**Note**:* indicates significance at p < 0.05. Abbreviations: η_p_², partial Etta squared; p value, probability value; min.km^-1^, minutes per kilometer; km.h^-1^, kilometer per hour; RPE, ratings of perceived exertion. The results revealed that men and women differed in overall performance (p **< 0.001), whereas** no significant sex-related interaction (p > 0.05: sex × time, sex × group, and group × time × sex) effects were found.
